# FABP4-mediated lipid droplet formation in *Streptococcus uberis*-infected macrophages supports host defence

**DOI:** 10.1186/s13567-022-01114-0

**Published:** 2022-11-12

**Authors:** Zhixin Wan, Shaodong Fu, Zhenglei Wang, Yuanyuan Xu, Yuanyuan Zhou, Xinguang Lin, Riguo Lan, Xiangan Han, Zhenhua Luo, Jinfeng Miao

**Affiliations:** 1 MOE Joint International Research Laboratory of Animal Health and Food Safety, Key Laboratory of Animal Physiology & Biochemistry, College of Veterinary Medicine, Nanjing Agricultural University, Nanjing, 210095 China; 2 Shanghai Veterinary Research Institute, Chinese Academy of Agricultural Sciences, Shanghai, 200241 China; 3 School of Water, Energy & Environment, Cranfield University, Cranfield, Bedfordshire, MK43 0AL UK

**Keywords:** FABP4, lipid droplets, *Streptococcus uberis*, bovine mastitis, inflammation

## Abstract

Foamy macrophages containing prominent cytoplasmic lipid droplets (LDs) are found in a variety of infectious diseases. However, their role in *Streptococcus uberis*-induced mastitis is unknown. Herein, we report that *S. uberis* infection enhances the fatty acid synthesis pathway in macrophages, resulting in a sharp increase in LD levels, accompanied by a significantly enhanced inflammatory response. This process is mediated by the involvement of fatty acid binding protein 4 (FABP4), a subtype of the fatty acid-binding protein family that plays critical roles in metabolism and inflammation. In addition, FABP4 siRNA inhibitor cell models showed that the deposition of LDs decreased, and the mRNA expression of *Tnf*, *Il1b* and *Il6* was significantly downregulated after gene silencing. As a result, the bacterial load in macrophages increased. Taken together, these data demonstrate that macrophage LD formation is a host-driven component of the immune response to *S. uberis*. FABP4 contributes to promoting inflammation via LDs, which should be considered a new target for drug development to treat infections.

**Supplementary Information:**

The online version contains supplementary material available at 10.1186/s13567-022-01114-0.

## Introduction

Bovine mastitis is the most economically important infectious disease in the dairy industry [1]. This condition is an inflammation of mammary tissues due to infection caused by specific pathogens. Bovine mastitis has a complex aetiology, and its multifactorial nature makes it difficult to control [2]. *Streptococcus uberis* is acknowledged as one of the most important environmental pathogens of mastitis worldwide. Large numbers of this bacterium are present in the environment and anatomic sites of cows [3]. There are a significant number of subclinical and clinical intramammary infections caused by *S. uberis* in both lactating and nonlactating cows [4]. Recent data indicate that the incidence of *S. uberis* infection is increasing globally [5].

Lipid droplets (LDs) were initially described as fat storage organelles in adipocytes but are increasingly recognized as dynamic players in lipid metabolism [6], with instrumental roles in diseases such as diabetes [7] and cancer [8] and in immune regulation, especially in the interactions between host and pathogens [9]. Parasites (such as trypanosomes [10] and *Plasmodium falciparum* [11]), bacteria (such as *Mycobacteria* [12] and *Chlamydia* [13]), and viruses (such as *hepatitis C* [14] and *dengue* [15]) induce and target LDs during their life cycles. The current view is that LDs support infection by providing microorganisms with substrates for effective growth. Pathogens require host-derived lipids to support their life cycles; LDs provide a source of these lipids. As a result, LDs have the potential to deliver effective host defences against intracellular pathogens. Triglyceride (TG) synthesis is essential for optimal inflammatory macrophage activation because its inhibition, which prevents LD development, has marked effects on the production of inflammatory mediators, including IL-1β, IL-6 and PGE2, and on phagocytic capacity [16]. Dias et al. indicated that LDs have important functions in the modulation of inflammatory mediator production in SARS-CoV-2-infected monocytes [17]. Our laboratory previously found that *S. uberis* infection significantly increases LDs in mammary epithelial cells, and these LDs appear to support pathogen infection [18]. Macrophages are the most consequential immune cells in mammary tissue, and the effect of LD levels on pathogen invasion and their specific mechanisms are unclear.

Fatty acid-binding protein 4 (FABP4), a subtype of the fatty acid-binding protein family, is a key transmitter of lipid metabolism and inflammation [19]. FABP4 has been identified as a cytosolic protein strongly upregulated during the differentiation of preadipocytes into adipocytes [20, 21]. Its abundance is positively correlated with free fatty acids, and high levels of FABP4 can directly damage endothelial cells. Conversely, injured endothelial cells can promote FABP4 levels due to the deposition of triglycerides, cholesterol and other lipid metabolic disorders [22]. FABP4 expression is readily detected in macrophages, particularly upon inflammatory activation [23]. Dou’s findings indicate that exogenous FABP4 interferes with adipocyte differentiation and induces p38/HSL-mediated lipolysis and p38/NF-κB-mediated inflammation in adipocytes in vitro and in vivo [24]. Hence, FABP4 plays critical roles in regulating inflammation and lipid metabolism. Xu’s results indicate that FABP4 contributes to renal interstitial fibrosis by promoting inflammation and lipid metabolism [22]. However, the specific role of FABP4 during infection is unknown.

Herein, we report that *S. uberis* infection causes a surge in LD levels in macrophages, accompanied by a dramatic increase in inflammation, and that regulating LD levels through the fatty acid synthesis pathway effectively controls inflammation and intracellular bacterial load, which may help decipher the molecular mechanisms involved in antimicrobial defence that could be exploited for the development of new anti-infective agents.

## Materials and methods

*Streptococcus uberis* (strain 0140J) was inoculated into Todd-Hewitt broth (THB) and incubated at 37 °C in an orbital shaker to log-phase growth (OD_600_ = 0.4–0.6). RAW264.7 cells were seeded at a density of 1 × 10^6^ cells for 6-well plates and then grown at 37 °C in Dulbecco’s modified Eagle’s medium (DMEM) with 10% foetal bovine serum (FBS, Gibco, USA). After reaching 70–80% confluency, the monolayers were treated with or without 40 µM BMS309403 (inhibitor of FABP4: Selleck Chemicals, Houston, TX, USA) for 24 h, 360 µM oleic acid (Sigma-Aldrich, USA) for 24 h or 10 µM C75 (inhibitor of fatty acid synthase: Sigma-Aldrich, USA) for 24 h, all at 37 °C, or transfected with 50 nM si*Fabp4* for 24 h at 37 °C using Lipofectamine 3000 reagent (Invitrogen). Transfection reagents and siRNA (si*Fabp4*) were purchased from Guangzhou Ruibo Biotechnology Co., Ltd. (Guangzhou, Guangdong, China). The sequence of si*Fabp4* was designed and listed as follows: CCACACCAGTCTCCTCAAT. The interference of the *Fabp4* gene was identified by qPCR and Western blotting (Figures 7A and B) in cell models. The treated cells were infected with *S. uberis* at a multiplicity of infection (MOI) of 10 for 3 h at 37 ℃ according to different test conditions. The supernatant and cells were collected separately and stored at −80 ℃ until use. Other reagents and materials are listed in Additional file 1. The effects of oleic acid and C75 on RAW264.7 cell viability are listed in Additional file 2.

### RNA extraction and quantitative real-time polymerase chain reaction (qPCR)

Total RNA was extracted by TRIzol reagent (TaKaRa, Dalian, China). Corresponding cDNA was obtained using reverse transcriptase and Oligo (dT) 18 primer (TaKaRa). An aliquot of the cDNA was mixed with 25 µL of SYBR^®^ Green PCR Master Mix (TaKaRa) and 10 pmol of each specific forward and reverse primer. All mixed systems were analysed in an ABI Prism 7300 Sequence Detection System (Applied Biosystems, Waltham, MA, USA). Relative gene expression was calculated using the method by Michael W. Pfaffl [25]. All primer sequences (Additional file 3) were synthesized by Tsinke Company (Beijing, China).

### Total protein extraction and Western blotting

Intracellular protein levels were determined by Western blotting. An anti-β-actin antibody (Bioworld, USA) was used as a loading control. Cells were washed twice in ice-cold phosphate buffered saline (PBS) and lysed by incubation in RIPA buffer (Beyotime, Nantong, China) containing PMSF (Beyotime, Nantong, China) on ice for 30 min. Supernatants were collected by centrifuging at 5000 × *g* for 10 min at 4 °C, and protein concentrations were determined using a Bicinchoninic Acid Assay Kit (Beyotime, Nantong, China) and detected with a spectrophotometer (Tecan, Männedorf, Switzerland). Proteins were separated by electrophoresis on a polyacrylamide gel and transferred to polyvinylidene fluoride membranes (Millipore, USA). The membranes were blocked with 5% nonfat milk diluted in Tris buffered saline with Tween-20 (TBST) for 4 h at room temperature (approximately 10–25 °C) and hybridized overnight with an appropriate primary antibody at 4 °C. Primary antibodies were diluted in TBST as follows: β-actin (1:10 000) and FABP4 (1:1000). The membranes were washed 3 times with TBST before and after incubation with horseradish peroxidase (HRP)-linked anti-rabbit IgG (CST, Massachusetts, USA, 1:10 000) secondary antibody at room temperature (approximately 10–25 °C) for 1 h. Signals were detected using an ECL Western Blot Analysis System (Tanon, Shanghai, China). Bands were quantified using ImageJ software (NIH, USA).

### Lipid droplet staining and counting

Lipid droplets were evaluated by staining RAW264.7 cells with BODIPY 493/503 to facilitate quantitation of neutral lipid content by flow cytometry [26]. Briefly, cells were quickly rinsed using 3 mL of PBS to remove media. After incubation with 2 µM BODIPY in the dark for 15 min at 37 °C, the cells were washed 3 times in PBS and detached. The cells were centrifuged at 400 × *g* for 5 min, resuspended in PBS, and immediately analysed by flow cytometry using a FACSCanto flow cytometer (BD, New Jersey, USA). Ten thousand cells per sample were analysed using CellQuest Pro acquisition and Flow Jo software.

### BODIPY staining and microscopy

The coverslips were washed with 3 mL of PBS 3 times and fixed with 4% formaldehyde for 15 min at room temperature. They were again washed 3 times with PBS after incubation with 2 µM BODIPY in the dark for 15 min at 37 ℃. They were then incubated with 10 µM DOPY in the dark for 10 min at room temperature and washed 3 times for 5 min in PBS. Finally, the plate was flooded with an anti-fluorescence quenching sealer and photographed with a laser confocal microscope [26].

### Assessment of biochemical parameters

The protein samples from RAW264.7 cells were extracted using cold lysis buffer and assayed using a BCA Protein Assay Kit. The levels of total cholesterol (TC), triglycerides (TGs), and nonesterified fatty acids (NEFAs) in cell lysates were measured using commercial kits according to the manufacturer’s instructions.

### Detection of relative enzyme activities

The activities of lactate dehydrogenase (LDH) and β-*N*-acetylglucosaminidase (NAG) in RAW264.7 cells were measured by commercial kits according to the manufacturer’s instructions.

### Viable bacterial count assay

Viable bacteria were enumerated as colony-forming units (CFU) on THB agar. CFUs were counted by the spread plate method after incubation for 12 h at 37 °C. RAW264.7 cells with or without oleic acid, C75, BMS309403 and si*fabp4* were incubated in DMEM with 10% FBS and plated at 80% confluence in 6-well plates. After infection with *S. uberis* for 3 h at mid-exponential phase (OD_600_ = 0.4–0.6), *S. uberis*-infected cells were washed 3 times (15 min at 37 °C each time) with PBS containing 100 mg/mL gentamicin, followed by gentamicin-free PBS. Cells were pelleted at 1.4 g for 10 min, both cells and the supernatant were collected, equal numbers of cells were lysed with sterile triple distilled water, and CFUs were counted by the spread plate method after incubation for 12 h at 37 °C [18].

### Flow cytometric analyses of bacterial burden

*Streptococcus uberis* was grown at 37 °C in THB medium until reaching an OD_600_ of 0.6. The medium was diluted to OD_600_ = 0.3 with fresh medium containing 0.1 mM FITC-d-Lys (Shengguang, Xiamen, China). The diluted bacteria were further incubated at 37 ℃ until an OD_600_ of 0.4–0.6 was achieved. The bacteria were centrifuged, washed with THB medium 3 times, and resuspended in cell culture medium. Cells were then incubated in 20 µmol FITC-d-Lys (a chemical biology approach that enables rapid and covalent incorporation and detection of a fluorescently derivatized peptidoglycan component during cell wall synthesis in real time) for 3 h at 37 ℃, washed 3 times in PBS, and detached with trypsin. Then, the cells were centrifuged at 400 × *g* for 5 min, resuspended in PBS, and immediately analysed by flow cytometry using a FACSCanto instrument; 10 000 cells/sample were analysed using CellQuest Pro acquisition software and FlowJo software.

### Immunofluorescence staining

Cell-filled slides were washed 3 times with PBS, fixed with 4% formaldehyde for 15 min, and washed an additional 3 times with PBS, and cell membranes were permeabilized with 0.5% PBST for 20 min. The slides were washed 3 more times with PBS before blocking with 5% goat serum for 2 h at room temperature. They were again washed 3 times with PBS and incubated with diluted primary antibody (1:100) overnight at 4 ℃. After 3 washes with PBS, secondary antibody (1:10 000) was added, and the slides were incubated for 2 h at room temperature. They were again washed 3 times with PBS and 10 μM DAPI for 10 min. After 3 washes with PBS, the plate was flooded with an anti-fluorescence quenching sealer and photographed with a laser confocal microscope.

### Statistical analyses

Statistical analyses were performed using GraphPad Prism 8 software. Sample numbers and repetitions are indicated in the figure legends. All data were analysed using an unpaired t test or one-way ANOVA as indicated in the figure legends. All data are represented as the means ± the standard deviation (SD) or standard error of the mean (SEM). For all experiments, *P* values < 0.05 were considered significant.

## Results

### The accumulation of LDs in macrophages associated with *S. uberis* infection

**Figure 1:**
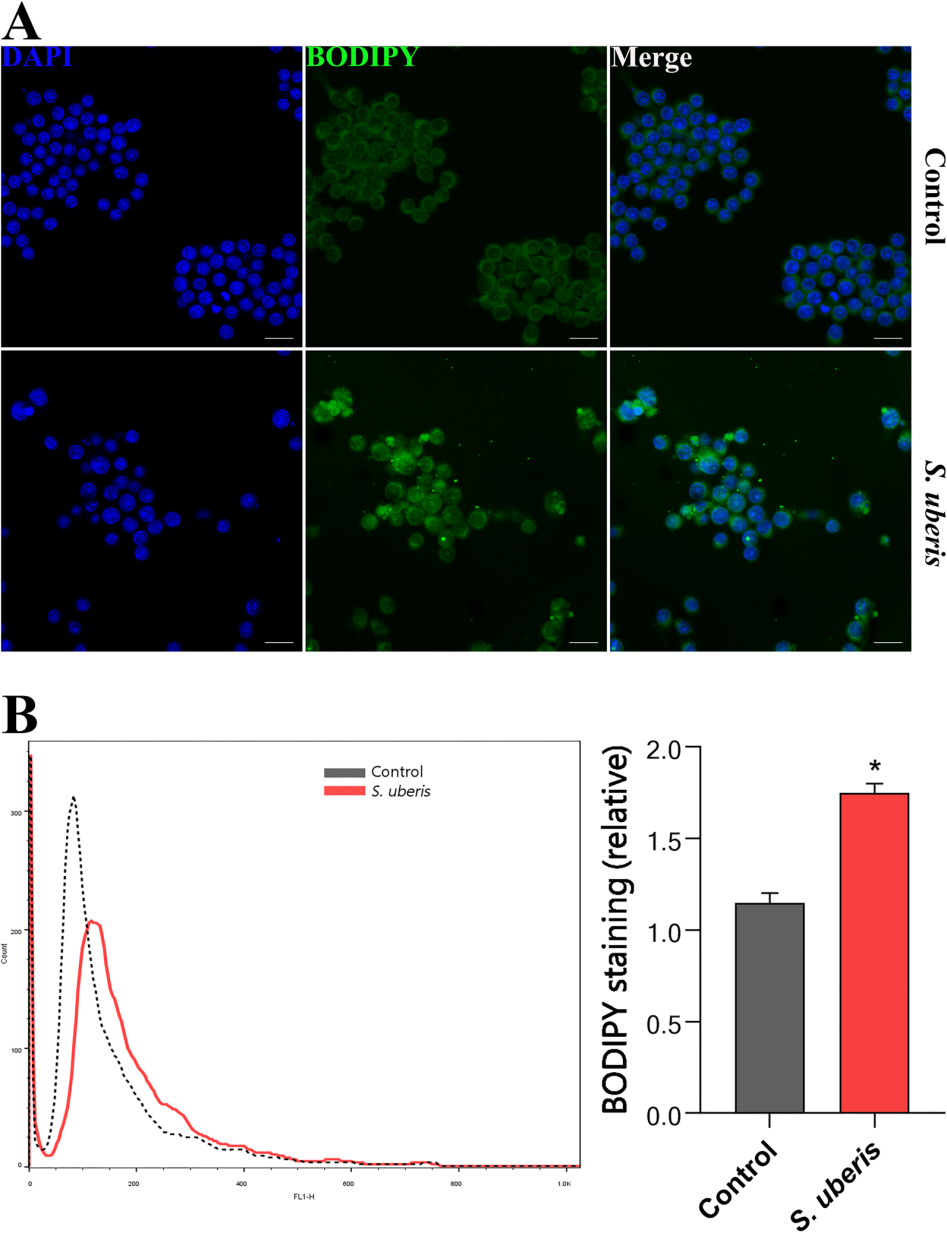
***S. uberis*** **infection leads to LD increases in macrophages**. RAW264.7 cells were infected with *S. uberis* at an MOI of 10 and incubated at 37 ℃ for 3 h as described in the “Materials and methods”. **A** BODIPY 493/503 staining for LD visualization (green) increased in *S. uberis* infection. RAW264.7 cells were fixed with 4% paraformaldehyde and stained with BODIPY. Uninfected cells were incubated under identical conditions. Images are representative of *n* = 10 samples/group. Scale bar, 20 µm. **B** Intracellular LD content was evaluated by staining cells (10 000/sample) with BODIPY 493/503, followed by analysis with CellQuest Pro acquisition software and FlowJo software. Data are presented as geometric mean fluorescence intensity (geoMFI). The dotted line indicates background fluorescence in uninfected cells. Experiment **B** is representative of 3 independent experiments. Data are presented as the means ± SEMs (*n* = 6, unless otherwise indicated). *(*P* < 0.05) = significantly different between the indicated groups by unpaired t test.

Host LDs are associated with various pathogen infections [27]. The fluorescent dye BODIPY493/503 was used for specific staining of intracellular LDs. Via laser confocal microscopy, we found that LDs in macrophages increased during *S. uberis* infection (Figure 1A). Furthermore, quantitative analysis via flow cytometry confirmed that *S. uberis* challenge significantly increased LD levels in macrophages (*P* < 0.05) (Figure 1B).

### *S. uberis* increases fatty acid synthesis in macrophages

**Figure 2:**
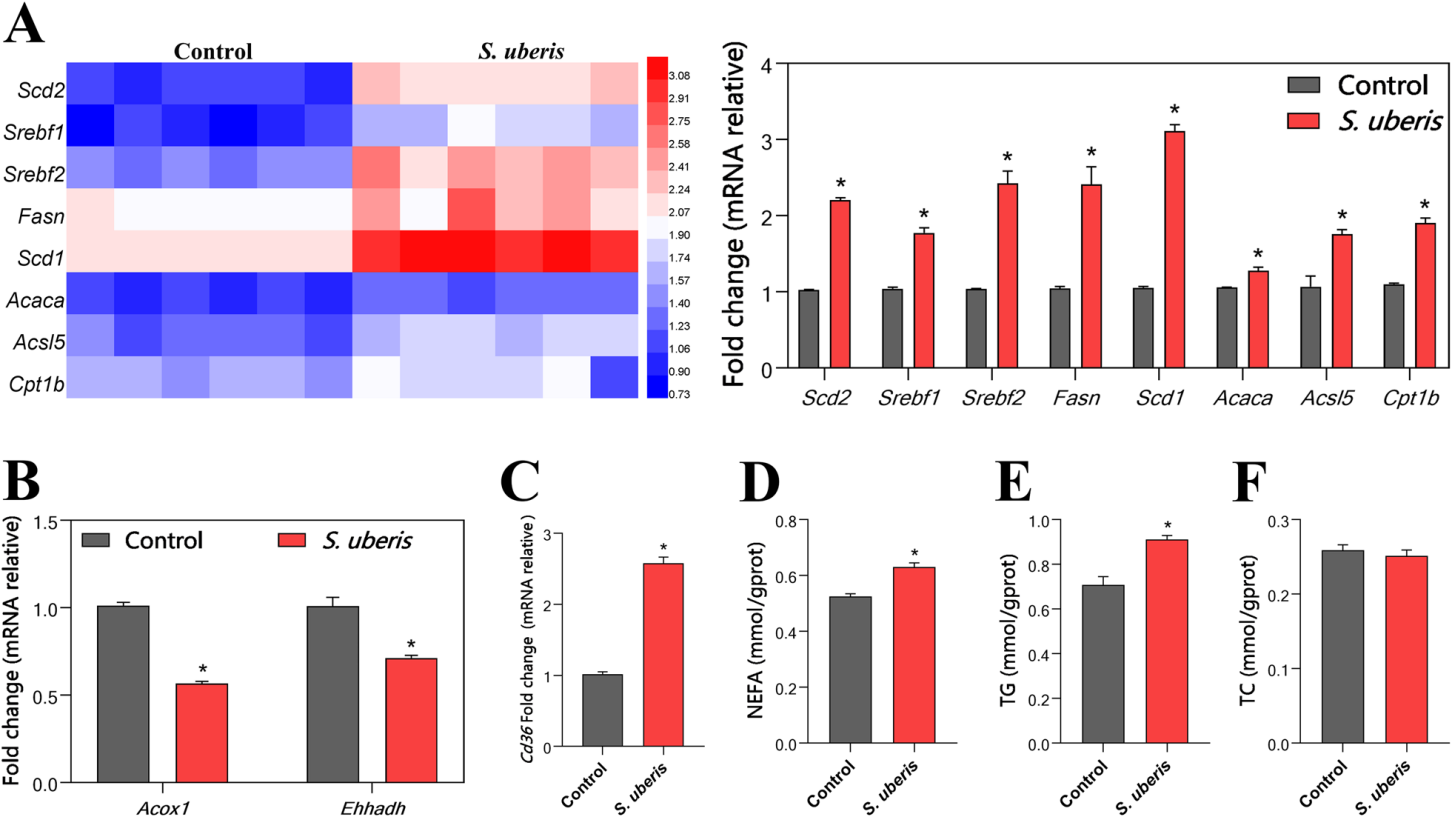
**Increased fatty acid synthesis in macrophages caused by S. uberis.** **A** Relative mRNA expression levels related to lipid synthesis in RAW264.7 cells during *S. uberis* invasion. Heatmap (on the left) and statistical chart (on the right). **B** *Acox1* and *Ehhadh* mRNA expression relative to that of fatty acid β oxidation. **C** *Cd36* mRNA relative expression related to fatty acid uptake. **D**–**F** NEFA, TG and TC contents in RAW264.7 cells during *S. uberis* challenge. Data are representative of 3 independent experiments. Data are presented as the means ± SEMs (*n* = 6, unless otherwise indicated). *(*P* < 0.05) = significantly different between the indicated groups by unpaired t test.

The level of LDs is closely related to lipid metabolism. Previous results in our laboratory have shown that *S. uberis* infection changes fatty acid synthesis pathways in mammary epithelial cells [18] and in mice [28]. We sought to confirm whether the bacteria have a similar effect on macrophages. By measuring the mRNA levels of multiple genes involved in fatty acid synthesis, we found that *S. uberis* significantly increased their expression to varying degrees (*P* < 0.05) (Figure 2A). The LD level is related not only to fatty acid synthesis but also to fatty acid oxidation and absorption of exogenous fatty acids. *S. uberis* infection significantly decreased the expression levels of genes related to the fatty acid oxidation pathway (*Acox1* and *Ehhadh*) (*P* < 0.05) (Figure 2B) and significantly upregulated the expression of *Cd36* (related to the absorption of exogenous fatty acids) (*P* < 0.05) (Figure 2C). To further verify that *S. uberis* causes increased fatty acid synthesis in macrophages, we examined NEFAs intracellular levels (Figure 2D), triglycerides (TGs) (Figure 2E) and total cholesterol (TC) (Figure 2F). Although TC did not show a significant change (*P* > 0.05), the NEFA and TG contents were significantly increased (*P* < 0.05) when challenged with *S. uberis*. Therefore, LD gains in macrophages caused by *S. uberis* may be related to the increase in fatty acid synthesis.

### Regulating lipid metabolism affects LDs in macrophages

**Figure 3:**
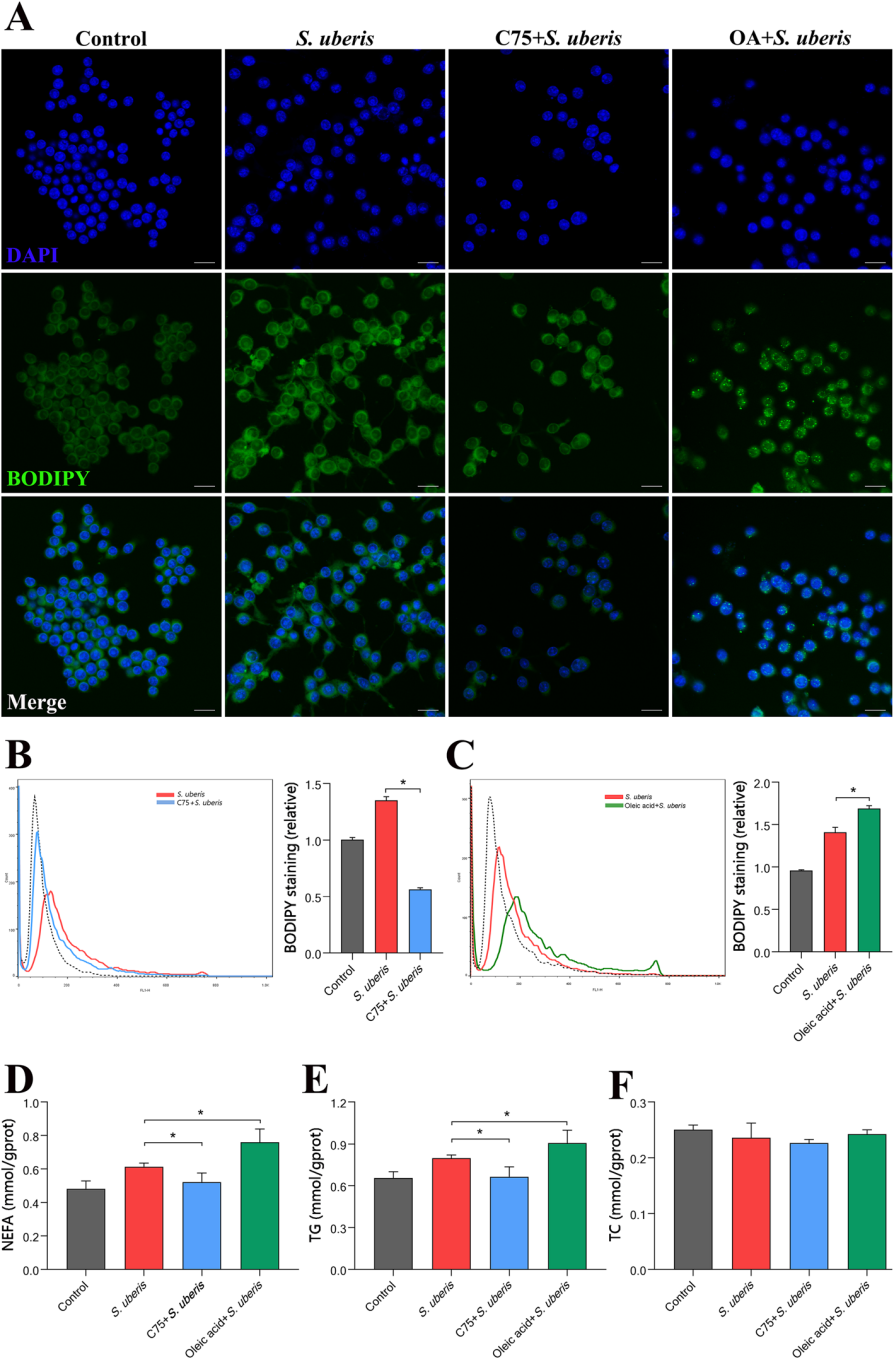
**Modulating lipid metabolism influences LD levels in macrophages.** RAW264.7 cells were treated with 10 µM C75 for 24 h to inhibit fatty acid synthesis, incubated with 360 µM oleic acid for 24 h, and infected with *S. uberis* in mid-exponential phase (MOI = 10) for 3 h at 37 ℃. **A** BODIPY 493/503 staining for LD visualization (green) in different groups. Images are representative of *n* = 10 samples/group. Scale bar, 20 µm. **B**, **C** Flow cytometry of BODIPY-stained RAW264.7 cells. Left: Representative histograms. Right: Mean fluorescence intensities from 3 independent experiments. The dotted line indicates background fluorescence in uninfected cells. Data are presented as the means ± SEMs (*n* = 6). *(*P* < 0.05) = significantly different between the indicated groups by one-way ANOVA. **D**–**F** NEFA, TG and TC contents in RAW264.7 cells during *S. uberis* challenge. Experiments **B**–**F** were conducted in triplicate. Data are presented as the means ± SEMs (*n* = 6). *(*P* < 0.05) = significantly different between the indicated groups by one-way ANOVA.

To further verify the relationship between LDs and lipid metabolism in macrophages, we used the fatty acid synthase (FASN)-specific inhibitor C75 to block de novo synthesis of fatty acids, and oleic acid was added to stimulate the formation of LDs. Confocal microscopy (Figure 3A) and flow cytometry (Figures 3B and C) data indicated that pretreatment with C75 ameliorated the increase in LDs caused by *S. uberis* infection (*P* < 0.05), while oleic acid markedly increased LD levels (*P* < 0.05). C75 pretreatment decreased NEFA (Figure 3D) and TG (Figure 3E) levels in macrophages (*P* < 0.05), while oleic acid significantly increased the levels of both (*P* < 0.05). For TC content (Figure 3F), there was no significant change between these groups (*P* > 0.05). Taken together, these results further demonstrate that fatty acid synthesis is closely related to LD levels and that in macrophages, LD contents can be regulated by affecting fatty acid metabolic pathways.

### Increased LDs reduce the *S. uberis* burden in macrophages

**Figure 4:**
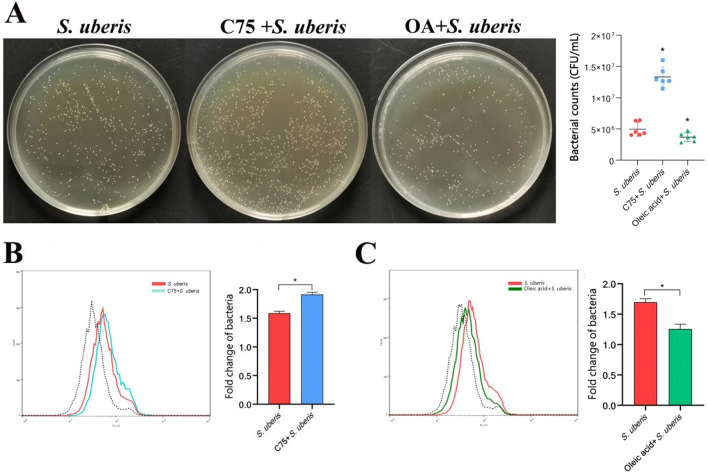
**Effect of LD levels on bacterial load in macrophages.** RAW264.7 cells were treated with 10 µM C75 for 24 h to inhibit fatty acid synthesis, incubated with 360 µM oleic acid for 24 h, and then infected with *S. uberis* in mid-exponential phase (MOI = 10) for 3 h at 37 ℃. **A** Viable bacteria enumerated as colony-forming units (CFU) on THB agar (left). CFUs were counted by the spread plate method after incubation for 12 h at 37 °C. The efficacy of C75 and oleic acid on the elimination of *S. uberis* was confirmed by culturing mMEC lysates on THB agar plates. The number of *S. uberis* colonies in cells (right). Data are presented as the means ± SDs (*n* = 6). *(*P* < 0.05) = significantly different between the indicated groups by one-way ANOVA. **B**, **C** Representative flow cytometry histogram of the FLI-H channel at 3 hpi in the FITC-d-Lys-*S. uberis*-infected RAW264.7 cells of the indicated groups. The dotted line indicates background fluorescence in the uninfected cells. Data are presented as the means ± SEMs (*n* = 6). *(*P* < 0.05) = significantly different between the indicated groups by one-way ANOVA. Experiments were conducted in triplicate.

LDs not only serve as organelles to store neutral fat but also play a crucial role in the interaction between pathogens and hosts [29]. Herein, we found that C75 and oleic acid significantly affected the bacterial load in macrophages while regulating LD levels in macrophages. The plate coating count of intracellular contents (Figure 4A) showed that C75 significantly increased the number of *S. uberis* colonies (*P* < 0.05), while oleic acid notably reduced the bacterial population (*P* < 0.05). To verify this conclusion, we used FITC-D-Lys fluorescent dye to stain *S. uberis*. Quantitative flow cytometry showed that C75 caused a significant increase in fluorescence intensity (*P* < 0.05) (Figure 4B), while oleic acid caused the opposite trend (*P* < 0.05) (Figure 4C). These results indicate that facilitating LD formation can effectively reduce the intracellular bacterial load and vice versa.

### Increased LDs result in excessive inflammation and cell damage in macrophages

**Figure 5:**
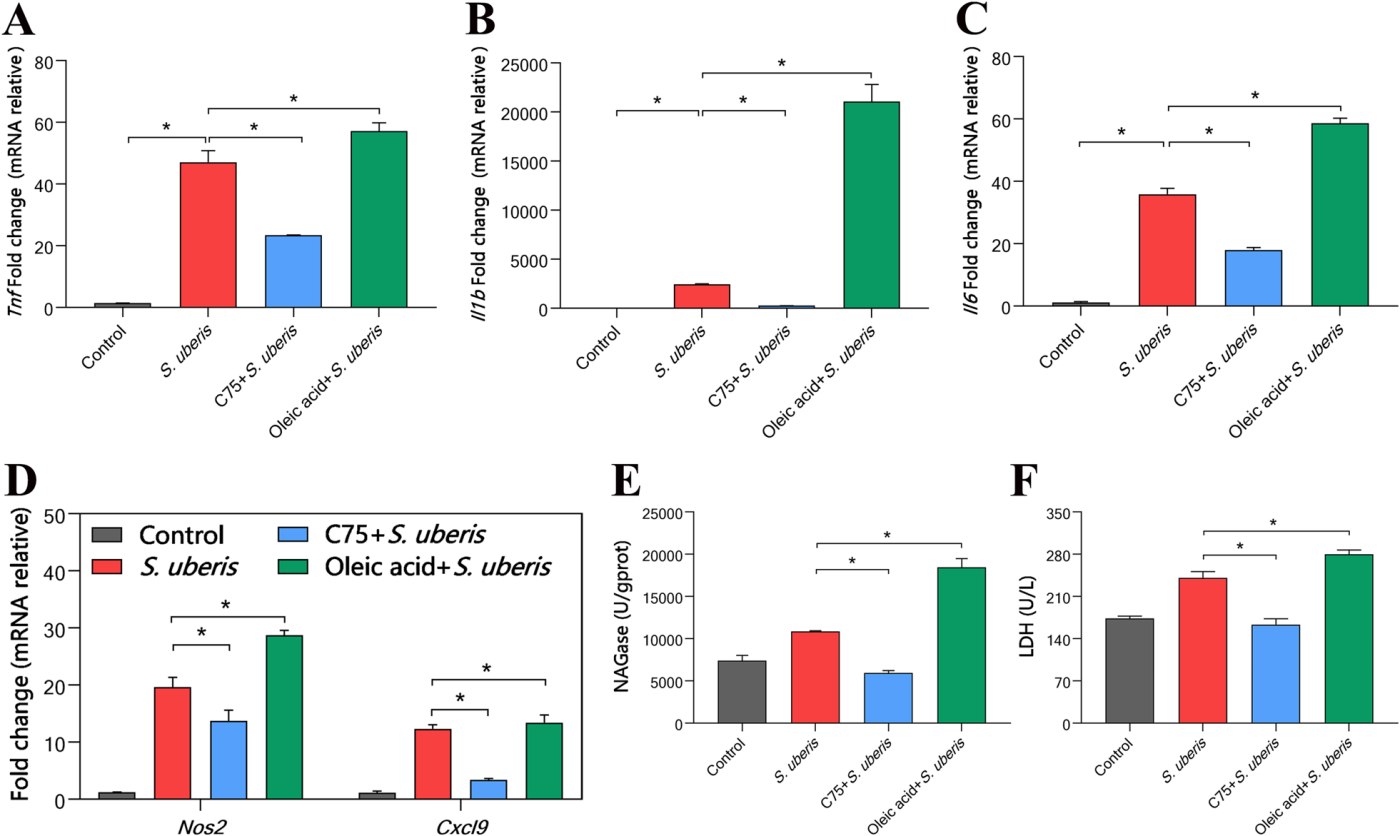
**LD increases in macrophages cause increased inflammation and cell damage.** RAW264.7 cells were treated with 10 µM C75 for 24 h to inhibit fatty acid synthesis and then incubated with 360 µM oleic acid for 24 h. The cells were then infected with *S. uberis* in mid-exponential phase (MOI = 10) for 3 h at 37 ℃. **A**–**C** *Tnf*, *Il1b*, and *Il6* mRNA expression. **D** *Nos2* and *Cxcl9* mRNA expression relative to inflammation. **E**, **F** NAG and LDH activity in RAW2647 cells. All experiments were repeated 3 times. Data are presented as the means ± SEMs (*n* = 6). *(*P* < 0.05) = significantly different between the indicated groups by one-way ANOVA.

A growing number of studies have shown that LDs not only play an important regulatory role in lipid homeostasis but also participate in inflammatory responses through different pathways [16]. We explored whether LDs exert an antimicrobial effect by affecting the expression of inflammatory mediators. By detecting the mRNA levels of multiple cytokines in macrophages, we found that *S. uberis* infection caused a sharp increase in the expression of *Tnf* (Figure 5A), *Il1b* (Figure 5B) and *Il6* (Figure 5C). *Il1b* increased nearly 4000 times (*P* < 0.05). C75 pretreatment significantly decreased mRNA levels (*P* < 0.05), while oleic acid supplementation increased the levels of inflammatory cytokines (*P* < 0.05). These results were confirmed by the detection of 2 other inflammation-related genes (Figure 5D). During infection, pathogens trigger the innate immune response. Excessive inflammation can cause dysregulation of homeostasis. By detecting the enzyme activity of the cell damage markers NAG (Figure 5E) and LDH (Figure 5F), we showed that C75 effectively reduced their activities (*P* < 0.05), while oleic acid pretreatment aggravated the cell damage caused by *S. uberis* (*P* < 0.05). Therefore, LDs induced by *S. uberis* participate in inflammatory responses in macrophages.

### FABP4 is responsible for LD accumulation during *S. uberis* challenge

**Figure 6:**
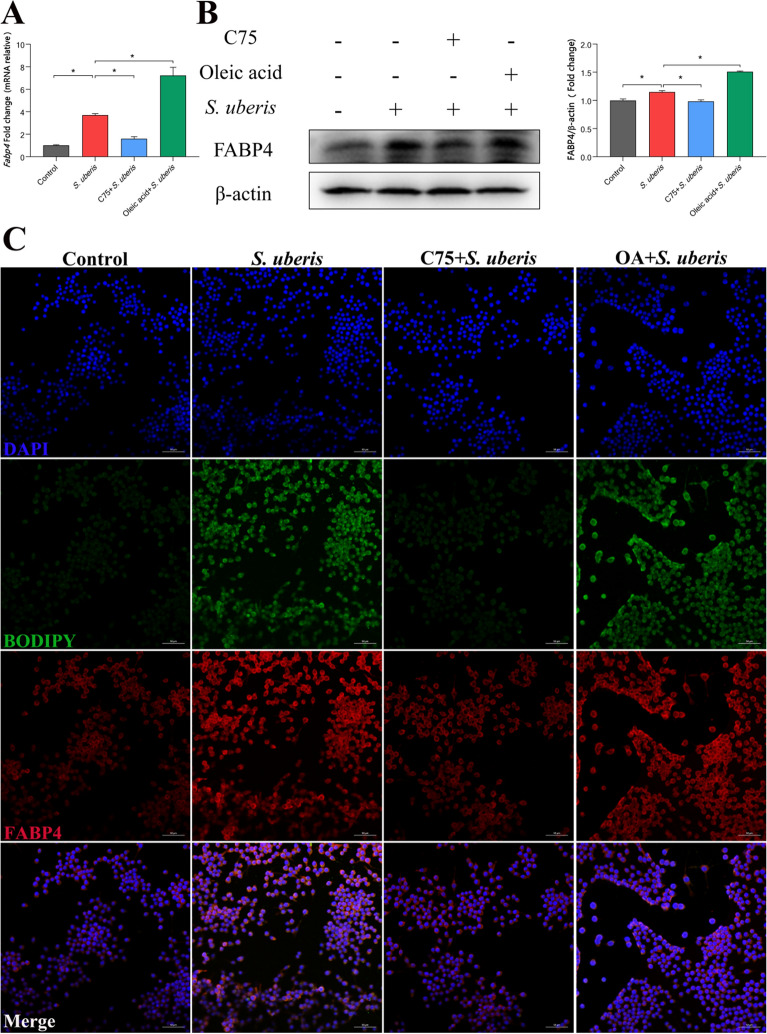
**FABP4 is involved in LD increases during S. uberis infection.** RAW264.7 cells were treated with 10 µM C75 for 24 h to inhibit fatty acid synthesis and then incubated with 360 µM oleic acid for 24 h. *S. uberis* in mid-exponential phase (MOI = 10) was added for 3 h at 37 ℃. **A** Relative *Fabp4* mRNA expression. **B** The protein expression levels of FABP4 with or without C75 or oleic acid during *S. uberis* infection were determined by Western blots in RAW264.7 cells. For quantitative analysis, bands were evaluated densitometrically with ImageJ analyser software normalized for β-actin density. **C** Immunofluorescence and LD staining were performed by FABP4 (red) and BODIPY (green) in the control group and stimulated *S. uberis* groups as well as the chemical pretreatment groups. Images are representative of *n* = 10 samples/group. Scale bar, 50 µm. Experiments **A**–**G** were repeated 3 times. Data are presented as the means ± SEMs (*n* = 6, unless otherwise indicated). *(*P* < 0.05) = significantly different between the indicated groups by one-way ANOVA.

To further explore the specific mechanism by which LDs and the inflammatory response are regulated in macrophages, we focused on the protein FABP4. FABP4 is not only involved in fatty acid synthesis but also participates in activation of the inflammatory response [30]. We found that *Fabp4* gene and protein expression was significantly increased during *S. uberis* infection (Figures 6A and B), C75 effectively downregulated expression (*P* < 0.05) and oleic acid significantly increased expression (*P* < 0.05). FABP4 protein and lipid droplets were specifically stained, and confocal microscopy showed (Figure 6C) that the expression of FABP4 increased with an increase in LDs in macrophages. These results suggest that FABP4 may mediate the formation of LDs in macrophages during *S. uberis* infection.

### FABP4 contributes to LD increases that ameliorate bacterial burden

**Figure 7:**
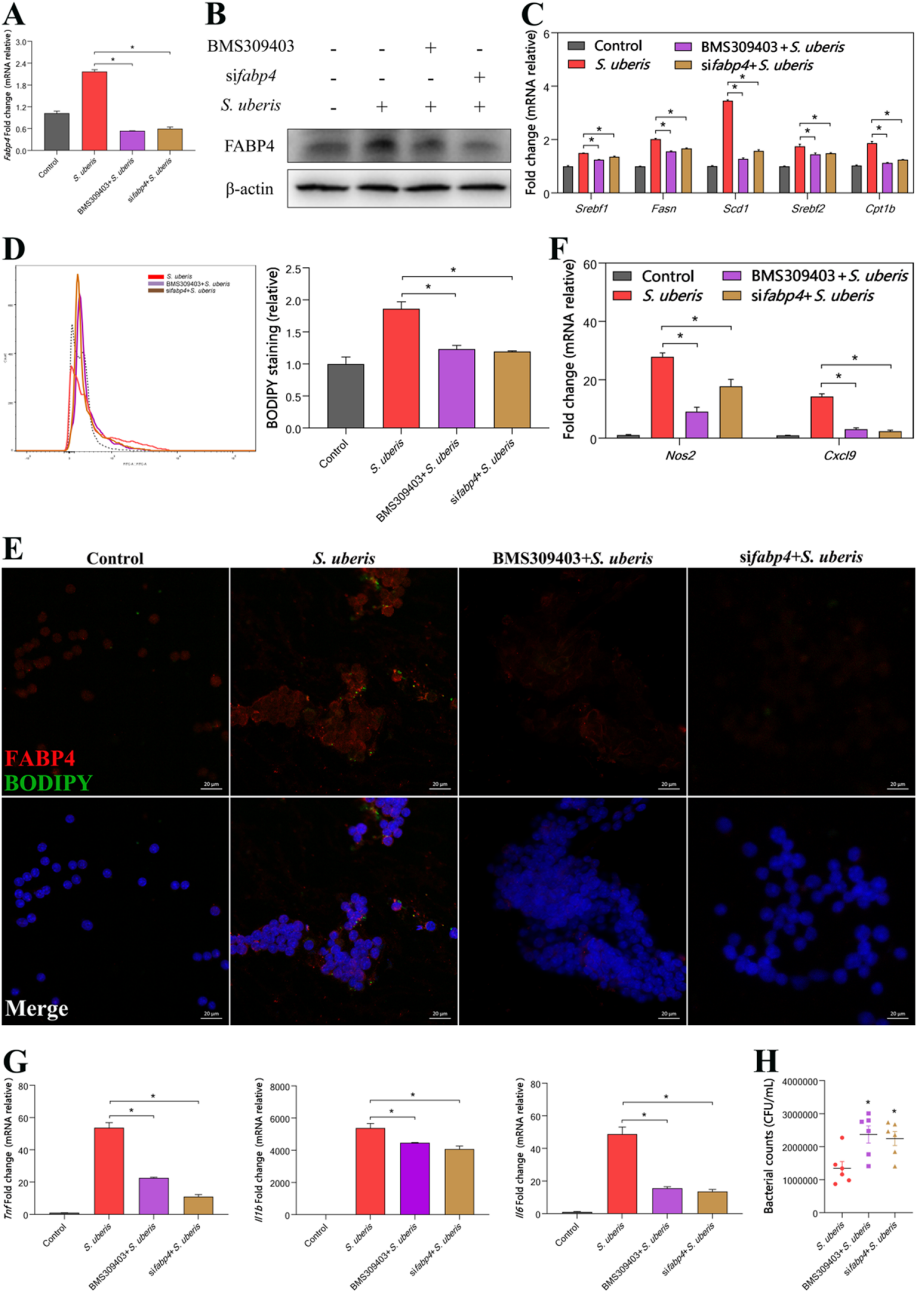
**Inhibiting FABP4 expression leads to reduced LDs and increased bacterial burden.** RAW264.7 cells were treated with 40 µM BMS309403 for 24 h or transfected with 50 nM si*Fabp4* for 24 h at 37 ℃ using Lipofectamine 3000 reagent (Invitrogen) to inhibit FABP4 expression prior to infection with *S. uberis* in mid-exponential phase (MOI = 10) for 3 h at 37 ℃. **A** *Fabp4* mRNA expression in the control and interference groups during *S. uberis* infection. **B** Protein expression levels of FABP4 with or without BMS309403 or si*Fabp4* during *S. uberis* infection were determined by Western blots of RAW264.7 cells. For quantitative analysis, bands were evaluated densitometrically with ImageJ analyser software normalized for β-actin density. **C** Relative mRNA expression of genes related to the synthesis of fatty acids. **D** Flow cytometry of BODIPY-stained RAW264.7 cells. Left: Representative histograms. Right: Mean fluorescence intensities from 3 independent experiments. The dotted line indicates background fluorescence in uninfected cells. Data are presented as the means ± SEMs (*n* = 6). *(*P* < 0.05) = significantly different between the indicated groups by one-way ANOVA. **E** Immunofluorescence and LD staining was performed by FABP4 (red) and BODIPY (green) in the control group and stimulated *S. uberis* groups as well as the chemical pretreatment groups. Images are representative of *n* = 10 samples/group. Scale bar, 20 µm. **F** Relative *Nos2* and *Cxcl9* mRNA expression. **G** *Tnf*, *Il1b*, and *Il6* mRNA expression. **H** The number of *S. uberis* colonies in the RAW264.7 cells after FABP4 or *Fabp4* treatment on THB agar plates. Data are presented as the means ± SDs (*n* = 6). *(*P* < 0.05) = significantly different compared with the *S. uberis* groups by one-way ANOVA. **A**–**D** and **F**–**H** experiments were repeated 3 times. Data are presented as the means ± SEMs (*n* = 6). *(*P* < 0.05) = significantly different between the indicated groups by one-way ANOVA.

To further verify that FABP4 is a target protein involved in regulating LD levels, we used small interfering RNA and the biochemical inhibitor BMS309403 to suppress FABP4 expression. si*Fabp4* and BMS309403 significantly reduced the increased expression of FABP4 caused by *S. uberis* (*P* < 0.05) (Figures 7A and B). Both treatments decreased the expression levels of genes related to fatty acid synthesis (*P* < 0.05) (Figure 7C). The results of flow cytometry (Figure 7D) and immunofluorescence (Figure 7E) showed that the level of LDs in macrophages was significantly decreased by reducing the expression of FABP4 (*P* < 0.05). The expression of inflammation-related genes in cells was also significantly decreased (*P* < 0.05) (Figures 7F and G). Correspondingly, the bacterial load in macrophages increased significantly when the expression of FABP4 was inhibited (*P* < 0.05) (Figure 7H). In conclusion, FABP4 promotes the synthesis of intracellular LDs, increases the inflammatory response and thus decreases the intracellular bacterial load.

## Discussion

Pathogenic infections alter host metabolism, often resulting in dysregulation of fatty acid metabolism and increased LDs [31]. The murine cell line RAW264.7 is a reasonable model, as it has been validated by others to show outputs similar to those of bovine macrophages when challenged with *S. uberis* [32, 33]. In this study, we found that *S. uberis* infection causes a dramatic increase in LDs in macrophages by increasing fatty acid synthesis. Promoting LD synthesis by regulating lipid metabolism effectively enhances inflammation and reduces intracellular bacterial load. Reducing FABP4 expression effectively decreases LD levels and inflammatory responses and leads to an increase in intracellular bacteria. This finding is consistent with research indicating that LDs play an important role in supporting host defences to fight pathogens [34]. These data indicate that FABP4 restrains *S. uberis* by promoting inflammation and LD increases, creating potential new drug targets for prevention and control of mastitis.

**Figure 8:**
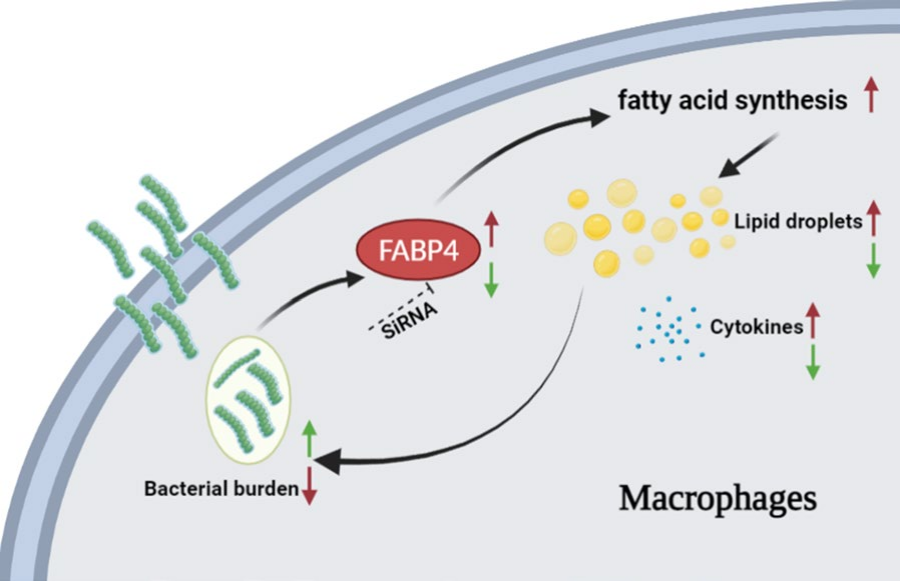
Model depicting the role of FABP4 mediated lipid droplets in macrophages after *S. uberis* infection

Macrophages are one of the most crucial immune cells in the mammary gland. Evidence suggests that LDs play a role in promoting immune responses [35]. Bosch et al. found that mammalian LDs are endowed with a protein-mediated antimicrobial capacity that is increased during polymicrobial sepsis and by LPS [34]. Knight et al. showed that an IFN-γ-driven, HIF-1α-dependent signalling pathway redistributes macrophage lipids into LDs and that macrophage LD formation is a host-driven component of the adaptive immune response to *M. tuberculosis* [36]. Herein, we found that an LD increase in macrophages occurs during *S. uberis* infection and that oleic acid-stimulated LD formation further upregulates inflammation and reduces the intracellular bacterial load. Moreover, inhibition of fatty acid synthase reduces LD levels and inflammation. These results further support the important role of LDs in host defence against infections (Figure 8).

FABP4, a member of the fatty acid-binding protein family, is involved in lipid metabolism and inflammation. This molecule is an important mediator of inflammatory processes in T cells, dendritic cells, and macrophages, as well as in experimental inflammation models [19, 37–39]. Recent studies have shown that FABP4 inhibitors can reduce lipid-induced ER stress-associated inflammation, ameliorate lipid deposition and suppress ROS and nuclear factor-kappa B (NF-κB) translocation [40]. Ge et al. have shown that cockroach antigen (CRA)-challenged FABP4-deficient mice exhibit attenuated eosinophilia and significantly reduced airway inflammation [30]. We found that FABP4 expression was significantly increased during *S. uberis* infection, and its expression levels were consistent with changes in intracellular LDs. Furthermore, silencing its expression by interfering with RNA or specific inhibitors effectively reduces the level of macrophage inflammation, thus increasing the amount of intracellular bacteria. These results suggest that FABP4 mediates LD synthesis to promote the inflammatory response of cells to resist invasion by *S. uberis*, although the exact mechanism requires further investigation.

LD formation occurs during the infection of macrophages with numerous intracellular pathogens, including *S. uberis*. A previous study in our laboratory found that *S. uberis* specifically induces LD formation as a pathogenic strategy to create a depot of host lipids for use as a carbon source to fuel intracellular bacterial growth in mammary epithelial cells [18]. Herein, we show that LD formation is not a bacterially driven process during *S. uberis* infection but rather occurs as a result of immune activation of macrophages as part of a host defence mechanism. This finding is consistent with data from *M. tuberculosis* infection in which macrophage LD formation is part of an adaptive immune response activated by IFN-γ [36]. Successful innate defence is critical for survival, and host species have efficiently coevolved with pathogens to develop a plethora of immune responses. As a vital source of nutrients for organisms in all eukaryotic cells, LDs store and supply essential lipids to produce signalling molecules, membrane building blocks, and metabolic energy. They are also significant players in pathogen and host defence. On the one hand, pathogens rob the host of nutrients to support their own intracellular growth and reproduction, and on the other hand, the host has evolved an immune response targeting LDs to fight infection. Macrophages are different from mammary epithelial cells in morphology and function. Macrophages are the main line of resistance to intracellular bacterial infection. Therefore, we hypothesize that LDs have evolved immune functions in macrophages that are distinct from those in epithelial cells, mainly playing a role in fighting rather than promoting infection.

In conclusion, our findings provide evidence for a role of FABP4 in LDs decreasing bacterial load by augmenting inflammation (Figure 8). Regulating lipid metabolism to fight bacteria exposes a number of therapeutic opportunities. We highlight that LDs constitute an intracellular first line of defence. The competition of the evolutionary strategy between microbes and host cells for LDs suggests that modulation of host metabolism may be able to limit pathogens, ultimately controlling infection. We also need to note the shortcomings of the research. In the current study, we only used one fatty acid (oleic acid), and possibly the results would vary if another fatty acid or a cocktail of fatty acids was chosen instead, which may be more reflective of what occurs in vivo.

## Supplementary Information

**Additional file 1.** **Key resources table.**

**Additional file 2. Effect of oleic acid and C75 on RAW 264.7 cell viability**. Cells were treated with oleic acid (A) or C75 (B) at the indicated concentrations for 24 h. Cell viability was measured by CCK8 assays.

**Additional file 3. Oligonucleotide sequences used for qPCR.**
