# Supplementary material for: FABP4-mediated lipid droplet formation in Streptococcus uberis-infected macrophages supports host defence
Source: Vet Res. 2022 Nov 12;53:90. doi: 10.1186/s13567-022-01114-0 (PMC9652580; doi:10.1186/s13567-022-01114-0)
Supplement: Supplementary file 1 — Additional file 1. Key resources table. [file 13567_2022_1114_MOESM1_ESM.docx]

**Additional file 3 Oligonucleotide sequences used for qPCR.**

| **Gene** | **Primers sequence (5′-3′)** | **Orientation** | **Efficiency** |
| --- | --- | --- | --- |
| ***Acaca*** | GATATCCCAGAGATGTTTCGGC | Forward | 2.11 |
|  | GTCAGCATGTCAGAAGGCAGAG | Reverse |  |
| ***Acox1*** | CGAGAAATCGAGAACTTG | Forward | 2.01 |
|  | CTTCGAGTGAGGAAGTTA | Reverse |  |
| ***Acsl5*** | TCAGTCATGACATTCTTCCGGGCA | Forward | 2.14 |
|  | CCAGCTTCACGTAATTGCAAGCCA | Reverse |  |
| ***Actb*** | TCTGGCACCACACCTTCTA | Forward | 2.05 |
|  | AGGCATACAGGGACAGCAC | Reverse |  |
| ***Cd36*** | CCTGGGAGTTGGCGAGAAA | Forward | 1.94 |
|  | CGATCACAGCCCATTCTCCT | Reverse |  |
| ***Cpt1b*** | GCTACCATGGGTGGATGTTTG | Forward | 2.00 |
|  | CGCTGAATTGTGGCTGACAC | Reverse |  |
| ***Cxcl9*** | TCTGCCATGAAGTCCGCTGTTCT | Forward | 2.33 |
|  | GTGGATCGTGCCTCGGCTGGT | Reverse |  |
| ***Ehhadh*** | CTTGGAATTCTGGATGTAG | Forward | 2.06 |
|  | TGGGTTTACCTATAACCG | Reverse |  |
| ***Fabp4*** | TCACCATCCGGTCAGAGAGTA | Forward | 2.07 |
|  | GCCATCTAGGGTTATGATGCTC | Reverse |  |
| ***Fasn*** | GATCCTGGAACGAGAACAC | Forward | 2.05 |
|  | AGACTGTGGAACACGGTGGT | Reverse |  |
| ***Il1b*** | AACCTGCTGGTGTGTGACGTTC | Forward | 2.14 |
|  | CAGCACGAGGCTTTTTTGTTGT | Reverse |  |
| ***Il6*** | CAAAGCCAGAGTCCTTCAGAG | Forward | 1.87 |
|  | GTCCTTAGCCACTCCTTCTG | Reverse |  |
| ***Nos2*** | AGCCTTGCATCCTCATTGG | Forward | 1.87 |
|  | CACTCTCTTGCGGACCATCT | Reverse |  |
| ***Scd1*** | CCGAAGTCCACGCTCGAT | Forward | 2.24 |
|  | TGGAGATCTCTTGGAGCATGTG | Reverse |  |
| ***Srebf1*** | GCGCTACCGGTCTTCTATCA | Forward | 2.12 |
|  | TGCTGCCAAAAGACAAGGG | Reverse |  |
| ***Srebf2*** | CCAAAGAAGGAGAGAGGCGG | Forward | 2.14 |
|  | CGCCAGACTTGTGCATCTTG | Reverse |  |
| ***Tnf*** | CATCTTCTCAAAATTCGAGTGACAA | Forward | 2.07 |
|  | TGGGAGTAGACAAGGTAGAACCC | Reverse |  |
