# Supplementary material for: FABP4-mediated lipid droplet formation in Streptococcus uberis-infected macrophages supports host defence
Source: Vet Res. 2022 Nov 12;53:90. doi: 10.1186/s13567-022-01114-0 (PMC9652580; doi:10.1186/s13567-022-01114-0)
Supplement: Supplementary file 2 — Additional file 2. Effect of oleic acid and C75 on RAW 264.7 cell viability. Cells were treated with oleic acid (A) or C75 (B) at the indicated concentrations for 24 h. Cell viability was measured by CCK8 assays. [file 13567_2022_1114_MOESM2_ESM.docx]

**Additional file 1 Key resources table**

| Reagent or Resource | Source | Identifier |
| --- | --- | --- |
| Antibodies | | |
| FABP4 | Abcam, Cambridge, UK | Cat#ab92501 |
| β-actin (4D3) monoclonal antibody | Bioworld, Nanjing, China | Cat#BS6007M |
| Anti-rabbit IgG, HRP-linked antibody | Cell Signaling Technology, Boston, USA | Cat#7074S |
| Anti-mouse IgG, HRP-linked antibody | Cell Signaling Technology, Boston, USA | Cat#7076 |
| Chemicals | | |
| Difluoro{2-[1-(3,5-dimethyl-2H-pyrrole-2-ylidene-N)ethyl]-3,5-dimethyl-1H-pyrrolato-N}boron (BODIPY 493/503) | Sigma-Aldrich, MO, USA | Cat#790389  CAS:121207-31-6 |
| FITC-d-Lys | Xiamen Shengguang Biological Technology Co., Ltd. | Cat#I0201 |
| C75 | Sigma-Aldrich, MO, USA | Cat#C5490  CAS:218137-86-1 |
| Oleic acid | Sigma-Aldrich, MO, USA | Cat#O1008  CAS:112-80-1 |
| BMS309403 | Selleck Chemicals, USA | Cat#S6622  CAS:300657-03-8 |
| Commercial Assays | | |
| Non-esterified fatty acid (NEFA) assay kit | Jiancheng, Nanjing, China | Cat#A042-2-1 |
| Total cholesterol (T-CHO) assay kit | Jiancheng, Nanjing, China | Cat#A111-1-1 |
| Triglyceride (TG) assay kit | Jiancheng, Nanjing, China | Cat#A110-1-1 |
| Lactate dehydrogenase (LDH) assay kit | Jiancheng, Nanjing, China | Cat#A020-2 |
| β-N-acetylglucosaminidase (NAG) assay kit | Jiancheng, Nanjing, China | Cat#A031-1-1 |
| Experimental Models: Bacterial Strains | | |
| *Streptococcus uberis* 0140 J | ATCC, Manassas, USA | Cat#ATCCBAA-854 |
| Experimental Models: Cell lines | | |
| Macrophage cell line (RAW264.7) | ATCC, Manassas, USA | Cat#TIB-71 |
| Software and Algorithms | | |
| GraphPad Prism v8 | GraphPad software | N/A |
| Image J v.2.0.0 | NIH | N/A |
| Flow Jo | Tree Star | N/A |
| Other |  |  |
| DMEMB(1X) | Gibco, New York, USA | Cat#11965-065 |
| 0.25% trypsin-EDTA (1X) | Gibco, New York, USA | Cat#25200-056 |
| Fetal bovine serum | Gibco, New York, USA | Cat#10099-141 |
| 4,6-diamidino-2-phenylindole (DAPI) | Sigma-Aldrich, MO, USA | Cat#10236276001 |
| Polyvinylidene fluoride membrane | Millipore, Bedford, USA | N/A |
