# Supplementary figures and images for: FABP4-mediated lipid droplet formation in Streptococcus uberis-infected macrophages supports host defence
Source: Vet Res. 2022 Nov 12;53:90. doi: 10.1186/s13567-022-01114-0 (PMC9652580; doi:10.1186/s13567-022-01114-0)

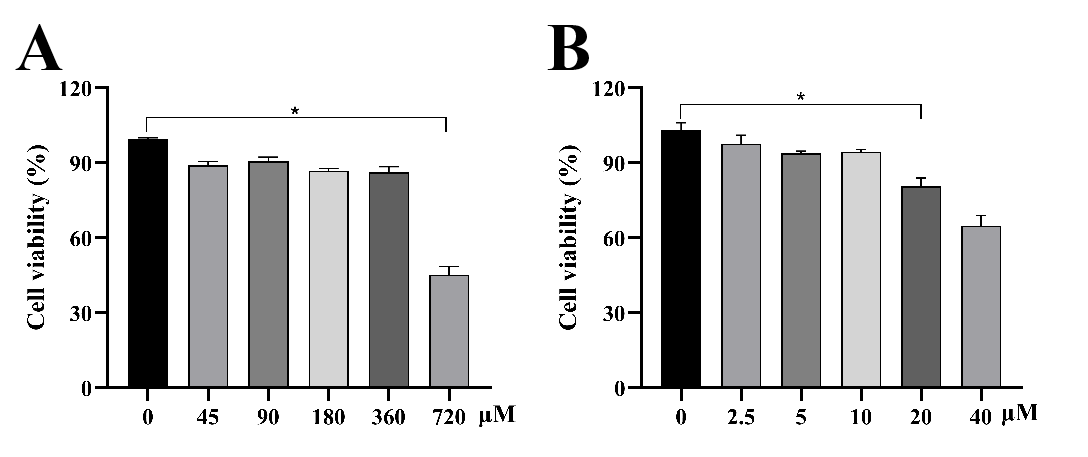

Supplement: Supplementary file 3 — Additional file 3. Oligonucleotide sequences used for qPCR. [file 13567_2022_1114_MOESM3_ESM.docx]
